# CT-based lung motion differences in patients with usual interstitial pneumonia and nonspecific interstitial pneumonia

**DOI:** 10.3389/fphys.2022.867473

**Published:** 2022-10-04

**Authors:** Jiwoong Choi, Kum Ju Chae, Gong Yong Jin, Ching-Long Lin, Archana T. Laroia, Eric A. Hoffman, Chang Hyun Lee

**Affiliations:** ^1^ Department of Internal Medicine, University of Kansas School of Medicine, Kansas City, KS, United States; ^2^ Department of Bioengineering, University of Kansas, Lawrence, KS, United States; ^3^ Department of Mechanical Engineering, University of Iowa, Iowa City, IA, United States; ^4^ Department of Radiology, Research Institute of Clinical Medicine of Jeonbuk National University-Biomedical Research Institute of Jeonbuk National University Hospital, Jeonbuk National University and Medical School, Jeonju, South Korea; ^5^ IIIHR-Hydroscience & Engineering, University of Iowa, Iowa City, IA, United States; ^6^ Department of Biomedical Engineering, University of Iowa, Iowa City, IA, United States; ^7^ Department of Radiology, University of Iowa, University of Iowa Hospitals and Clinics, Iowa, IA, United States; ^8^ Department of Radiology, Seoul National University College of Medicine, Seoul National University Hospital, Seoul, South Korea

**Keywords:** interstitial lung disease, idiopathic pulmonary fibrosis, usual interstitial pneumonia, computed tomography, lung motionography, quantitative computed tomography image matching, image registration, computational biomechanics

## Abstract

We applied quantitative CT image matching to assess the degree of motion in the idiopathic ILD such as usual interstitial pneumonia (UIP) and nonspecific interstitial pneumonia (NSIP). Twenty-one normal subjects and 42 idiopathic ILD (31 UIP and 11 NSIP) patients were retrospectively included. Inspiratory and expiratory CT images, reviewed by two experienced radiologists, were used to compute displacement vectors at local lung regions matched by image registration. Normalized three-dimensional and two-dimensional (dorsal-basal) displacements were computed at a sub-acinar scale. Displacements, volume changes, and tissue fractions in the whole lung and the lobes were compared between normal, UIP, and NSIP subjects. The dorsal-basal displacement in lower lobes was smaller in UIP patients than in NSIP or normal subjects (*p* = 0.03, *p* = 0.04). UIP and NSIP were not differentiated by volume changes in the whole lung or upper and lower lobes (*p* = 0.53, *p* = 0.12, *p* = 0.97), whereas the lower lobe air volume change was smaller in both UIP and NSIP than normal subjects (*p* = 0.02, *p* = 0.001). Regional expiratory tissue fractions and displacements showed positive correlations in normal and UIP subjects but not in NSIP subjects. In summary, lung motionography quantified by image registration-based lower lobe dorsal-basal displacement may be used to assess the degree of motion, reflecting limited motion due to fibrosis in the ILD such as UIP and NSIP.

## 1 Introduction

Idiopathic pulmonary fibrosis (IPF) is a chronic, progressive fibrosing interstitial pneumonia of unknown cause in adults characterized by the progressive worsening of dyspnea and lung function and is associated with poor prognosis. CT features of fibrosis and honeycombing are strongly correlated with forced vital capacity (FVC) and diffusion capacity of carbon monoxide (DL_CO_) measurements (Lynch et al., 2005). It has been known that the extent of fibrosis and honeycombing on CT is predictive of survival in IPF (Flaherty et al., 2003; Jeong et al., 2005; Best et al., 2008; Shin et al., 2008; Sumikawa et al., 2008; Raghu et al., 2018, 2022).

Elastography is a technique that uses the fact that a pathological process alters the elastic properties of the involved tissue or organ and is applied for the non-invasive evaluation of the stiffness of a lesion in the breast and fibrosis in the liver (Goddi et al., 2012; Barr et al., 2015). The degree of fibrosis in the lung parenchyma is also critical for the survival, prognosis, and treatment of interstitial lung disease (ILD) (Raghu et al., 2022). Elastography has been used with ultrasonography and MRI for the breast and liver; however, the lung parenchyma is not easily evaluated with this application due to the presence of air and respiratory motion artifacts. In the lung, the local lung motion can be computed using the image registration of volumetric CT images at different lung volumes (Yin et al., 2009; Choi, 2011; Yin et al., 2013; Jahani et al., 2014; Jahani et al., 2015; Jahani et al., 2017; Shin et al., 2020; Kang et al., 2021).

Recent advances in quantitative CT imaging and image matching techniques enabled the utilization of local lung information of inspiration and expiration CT scans in the computation of changes between the scans (Choi et al., 2010; Yin et al., 2010; Galban et al., 2012). Quantitative analysis of regional lung structures and functions of airway segments and lung parenchyma have been utilized to assess the regional lung characteristics, mostly for obstructive lung diseases such as asthma and chronic obstructive pulmonary disease (COPD) (Chae et al., 2020; Choi J et al., 2017; Choi et al., 2013; Choi et al., 2015; Galban et al., 2012; Jahani et al., 2017). Attempts to utilize these quantification methods for ILD are also increasing (Flaherty et al., 2003; Jeong et al., 2005; Best et al., 2008; Shin et al., 2008; Sumikawa et al., 2008). A mass preserving non-rigid image registration technique (Yin et al., 2009) can provide the matching of local lung regions between CT images at two different lung volumes. The method has been utilized to compute the regional displacement of local lung parenchyma between inspiration and expiration (Yin et al., 2013). The recent application of the cross-volume (inspiratory-expiratory) CT image matching-derived three-dimensional (3D) lung motion map differentiated regional lung motions between supine and prone positing in healthy subjects (Shin et al., 2020) and impaired diaphragm motion in patients with COPD and idiopathic pulmonary fibrosis (IPF) compared to healthy controls (Kang et al., 2021).

In this study, we applied this local lung “motionography” information based on cross-volume image matching technique to investigate if it can differentiate between the usual interstitial pneumonia (UIP) and nonspecific interstitial pneumonia (NSIP).

## 2 Materials and methods

### 2.1 Patient selection and data acquisition

This study was retrospectively designed and approved by the institutional review board, and informed consent was waived. From January 2013 to December 2017, we retrieved 361 idiopathic interstitial pneumonia (IIP) patients who underwent CT scans and pulmonary function tests (PFTs) from the hospital information system. Two chest radiologists (K.J.C. and C.H.L. with 5 and 20 years of experiences, respectively) reviewed CT images with four categories (UIP, probable UIP, indeterminate UIP, and alternative diagnosis) for UIP diagnosis according to the ATS/ERS guideline in consensus (Raghu et al., 2018). Twenty patients with definite UIP pattern on the CT scan and 11 patients with pathologic UIP were regarded as IPF on multidisciplinary diagnosis (age = 71.6 ± 6.7, M:F = 19:12). Pathologic confirmed NSIP patients were included as the NSIP group (age = 61.9 ± 8.6, M:F = 3:8) (Supplementary Figure S1). For the comparison, data of additional 21 normal subjects (age = 58.0 ± 13.4, M:F = 10:11) who participated in a previous study (Kim et al., 2017) were included in our analysis. The normal subjects had normal PFT results, normal chest CT scans, and no known history of lung disease or surgery.

Imaging was performed using a 128 multi-detector CT scanner (Ingenuity, Philips Healthcare, Best, Netherlands) under full inspiration and full expiration of the patients. The patients were coached by the radiology technician to take a full inhalation and full exhalation, respectivley. CT parameters were as follows: 120 kVp tube voltage, 170 reference mAs tube current-time product, z-dome, 3D dose modulation, 1.0 mm slice thickness, 1.0 mm reconstruction increment, YC 0 reconstruction filter, 0.5 s rotation time. The average voxel volume was 0.294 mm^3^. PFTs were performed according to the American Thoracic Society (ATS)/European Respiratory Society (ERS) guidelines (Miller et al., 2005). Dynamic study was first done. Then, static lung volumes were measured, followed by a bronchodilator test. Finally, DL_CO_ was measured. From the spirometry data, forced expiratory volume in 1 s (FEV_1_), FVC, and the FEV_1_/FVC ratio were assessed.

### 2.2 Inspiratory and expiratory CT image segmentation and registration

Supplementary Figure S2 shows the flow chart to compute the displacement at local lung regions from a pair of volumetric CT images at inspiration and expiration. First, individual volumetric CT images at inspiration and expiration were segmented and measured for the airway, vessels, lungs, and lobes, utilizing Apollo 2.0 (VIDA Diagnostics, Coralville, Iowa, United States). Then, a mass preserving non-rigid image registration method (Yin et al., 2009) was employed to obtain the local-to-local image matching between inspiration and expiration. The method determines a spatial transformation that matches the two images by minimizing a cost function that is the sum of the squared tissue volume difference (SSTVD). The cost function serves to minimize the local tissue volume difference within the lungs between matched regions, preserving the tissue mass of the lungs if the tissue density is assumed to be constant in the lung. This is particularly appropriate because air changes in the lung while the change of tissue components is minimal. The multiresolution approach adds the quality of local lung matching.

### 2.3 Local lung displacement

From matched local lung parenchymal units at a sub-acinar scale, 3D displacement vectors from expiration to inspiration were computed by the voxel-wise subtraction of the position vector on expiratory CT ***x*** ^EX^ from the matched position vector on inspiratory CT ***x*** ^IN^, where ***x***, EX, and IN denote the position vector, expiratory CT, and inspiratory CT, respectively. Two-dimensional (2D) dorso-basal (DB) displacements were also computed using only dorso-ventral and apico-basal components and neglecting transverse changes. 3D displacements and DB displacement magnitudes were both normalized by the cubic root of the global lung volume change from expiration to inspiration CT scans (ΔV) (Eq. 1), in order to reduce the effect of inter-subject variability by inspiration and expiration lung volumes in quantification. Normalized 3D local displacement was denoted by *s* ^∗^

$$s^{*}=s/\;\sqrt[{3}]{\Delta V\;}=\left|x^{IN}-x^{EX}\right|/\;\sqrt[{3}]{\Delta V\;}$$ (1)

DB displacement that excludes the transverse direction was denoted by *s*_yz_^*^, where the *y* and *z* directions are ventral to the dorsal and apical to basal directions, respectively, as indicated in Supplementary Figure S3. From DB vector, the displacement angle from y axis, *θ*, was computed. For visual interpretation, 3D displacement vectors were plotted on inspiration image color-coded by *s* ^*^.

### 2.4 Other functional imaging metrics

As imaging-based large-scale functional indicators, air volumes in the whole lung and the lobes were measured on inspiration and expiration. Mean local tissue fractions (TFs) on inspiration and expiration, which may also indicate the degree of fibrosis, were computed and compared with displacement variables. TFs in individual parenchymal units were averaged in the five lobes and combined regions.

### 2.5 Statistical analysis

Student’s t-tests were conducted for comparison between the groups of normal and ILD patients. When categorizing the ILD patients into NSIP and UIP, and comparing between the groups of normal, NSIP, and UIP, the analysis of variance (ANOVA) was done along with Tukey’s post hoc test to determine significant pairwise comparisons. Statistical analyses were performed using the R statistical programming environment, version 3.0.2 (the R Foundation, Vienna, Austria). *p* values <0.05 were considered significant.

## 3 Results

### 3.1 Global and regional lung functions

**FIGURE 1:**
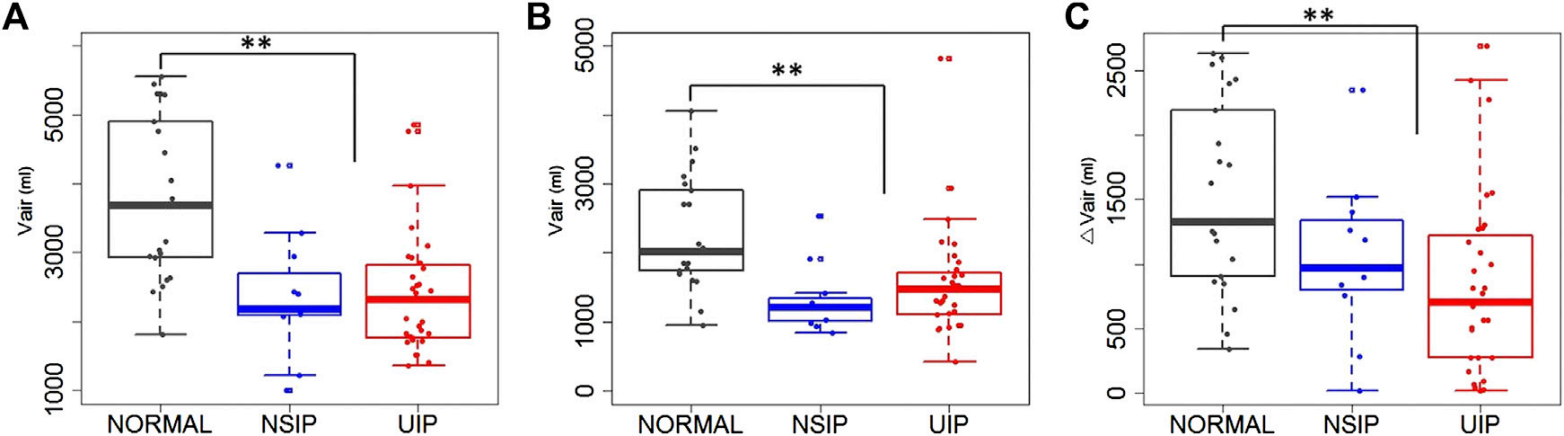
Air volumes at **(A)** inspiration and at **(B)** expiration, and **(C)** air volume changes between inspiration and expiration in the whole lung of normal, NSIP, and UIP subjects. ** denotes *p* < 0.05.

Lung function test values and CT-based volumetric parameters were compared between normal, NSIP, and UIP subjects. ILD (both NSIP and UIP) patients were differentiated from normal subjects. In ILD, FEV_1_ and FVC were decreased (*p* < 0.001, both), while FEV_1_/FVC was not different. DL_CO_ was also decreased (*p* = 0.001) (Table 1). The static air volumes at inspiration were decreased in the whole lung (*p* < 0.001), the upper lobes (*p* = 0.011), and the lower lobes (*p* < 0.001). In regard to the dynamics between inspiration and expiration, the air volume change was decreased in the whole lung (*p* < 0.001) and in the lower lobes (*p* < 0.001) but not in the upper lobes (Figure 1). Noticeably, only FVC differentiated between NSIP and UIP within ILD and no other parameters in PFT or volumetric lung did. Only the inter-subject variability of the upper lobe air volumes was found greater among UIP subjects than normal or NSIP subjects.

**TABLE 1:** Comparison of the lung function test results and volumetric parameters between normal, NSIP, and UIP patients.

	Normal (*n* = 21)	NSIP (*n* = 11)	UIP (*n* = 31)	NSIP + UIP vs. Normal^a^	NSIP vs. Normal^b^	UIP vs. Normal^b^	UIP vs. NSIP^b^
FEV_1_ (%pred)	110.1 ± 16.2	97.7 ± 20.0	84.9 ± 21.0	< 0.001	0.230	< 0.001	NA
FEV_1_/FVC (%pred)	80.9 ± 4.6	80.5 ± 3.9	83.1 ± 7.6	0.290	NA	NA	NA
FVC (%pred)	99.6 ± 13.5	87.4 ± 18.7	69.3 ± 16.6	< 0.001	0.120	< 0.001	0.009
DL_CO_ (%pred)	86.6 ± 24.3	66.9 ± 15.6	57.1 ± 21.4	0.001	0.130	0.003	0.420
Air volume, IN, whole (ml)	3792 ± 1194	2373 ± 906	2415 ± 894	< 0.001	0.001	< 0.001	NA
Air volume, IN, upper (ml)	1993 ± 639	1644 ± 559	1516 ± 564	0.011	NA	0.0160	NA
Air volume, IN, lower (ml)	1799 ± 596	729 ± 396	899 ± 430	< 0.001	< 0.001	< 0.001	NA
Air volume change, upper (%TLC)	19.2 ± 6.1	19.3 ± 9.9	16.3 ± 7.6	0.270	NA	NA	NA
Air volume change, lower (%TLC)	27.6 ± 8.1	15.2 ± 7.1	15.8 ± 6.7	< 0.001	< 0.001	< 0.001	NA

a *p* –values of Student’s t-test.

b *p* –values of ANOVA post hoc test (NA stands for “not applicable” and denotes p > 0.05 by ANOVA).

### 3.2 Displacement magnitude and vectors

**FIGURE 2:**
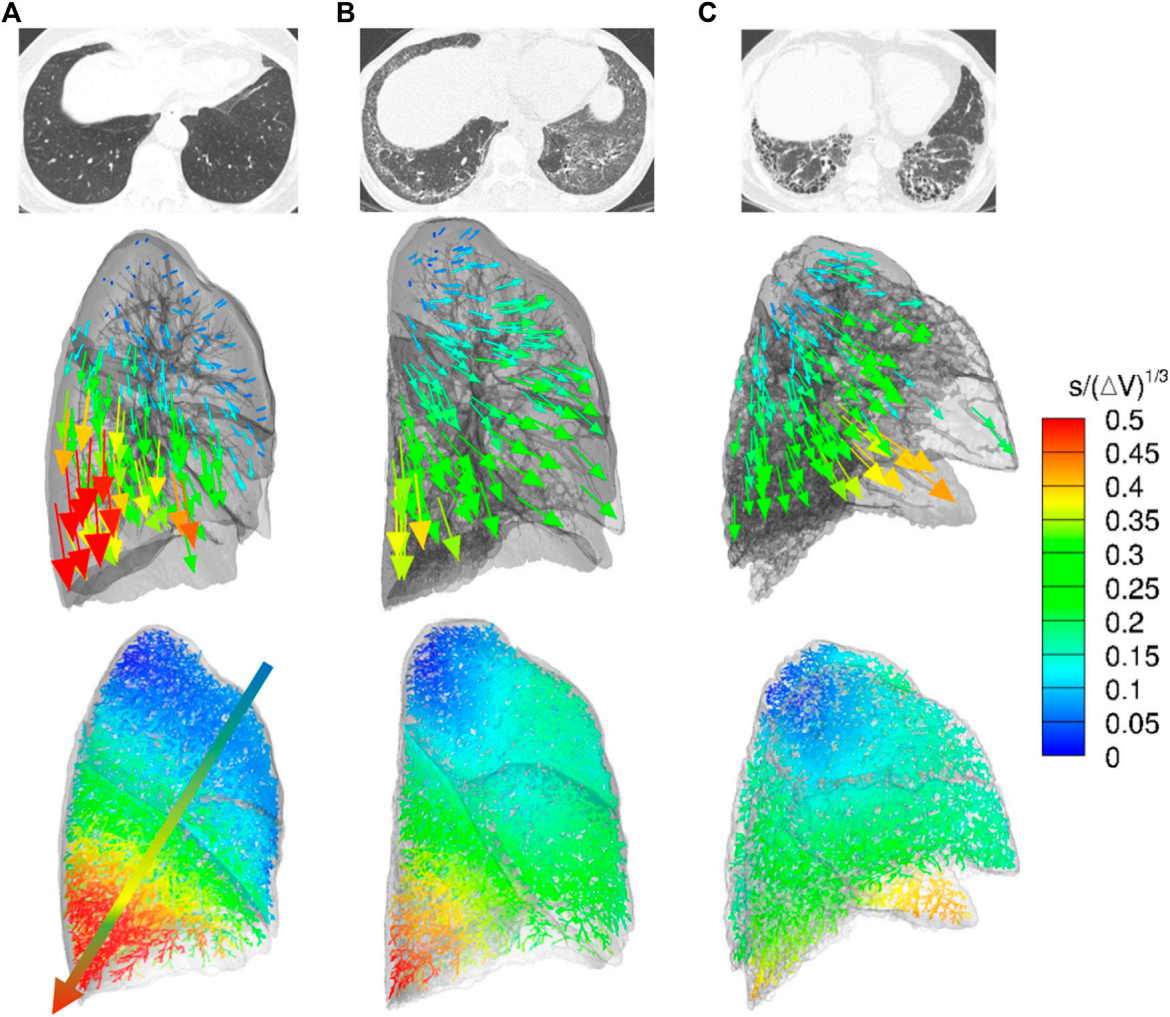
CT axial views (top), displacement vectors from expiration to inspiration (middle), and normalized dorsal and basal displacement magnitude maps on the entire conducting airway model (bottom) in representative **(A)** normal, **(B)** NSIP, and **(C)** UIP subjects.

Local lung displacements are presented for the normal, NSIP, and UIP subjects in Figure 2, demonstrating a hundred DB displacement vectors and magnitude maps in the entire conducting airway models, as well as CT axial views. In the normal subject, the local displacement of the lower lobe near the posterior costophrenic angle was the highest with the dorso-basal distribution. We speculated that the DB displacement is greater in the more gravitationally dependent lung regions and directions of the displacement vectors reflected regional lung deformation characteristics, similar to regional relative ventilation. In the UIP pattern, the local displacement of the lower lobe was significantly decreased near the posterior costophrenic angle and basal lung, which may represent the advanced fibrosis showing limited lung motions in the lung parenchyma. Lung regions moved more in the ventral direction, which could be attributable to a significant decrease in the movement toward the basal direction. The NSIP subject shows the intermediate characteristics of the displacement distribution toward the posterior costophrenic angle in between the normal and UIP subjects.

### 3.3 Displacement and angle

**FIGURE 3:**
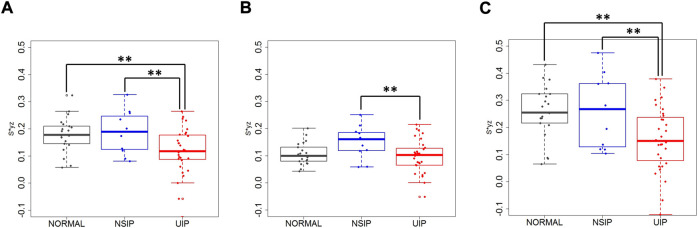
Dorsal basal displacement in **(A)** the whole lung, **(B)** the upper lobes, and **(C)** the lower lobes of normal, NSIP, and UIP subjects. ** denotes *p* < 0.05.

Local lung displacements between inspiration and expiration for all 3D components and for DB motions were compared in the whole lung, the upper lobes, and the lower lobes between normal, NSIP, and UIP patients (Table 2; Figure 3). DB (2D) relative displacement magnitudes in the lower lobes were found smaller in UIP lungs than not only normal lungs (*p* = 0.007) but also NSIP lungs (*p* = 0.036). The local displacement magnitude of the lower lobes near the posterior costophrenic angle noticeably decreased in the UIP subjects than NSIP (*p* = 0.040). ILD patients had a decreased DB displacement angle, *θ*, in the whole lung (*p* = 0.008) and in the lower lobes (*p* < 0.001) than normal subjects. UIP subjects show the same results that *θ* decreased in the whole lung (*p* = 0.012) and in the lower lobes (*p* < 0.001), which reflects limited lung motions toward the basal (z) direction. In the upper lobes, DB displacement increased in NSIP lungs than in UIP lungs (*p* = 0.032), while it is similar between normal and UIP lungs.

**TABLE 2:** Comparison of the motionographic variables between normal, NSIP, and UIP patients.

	Normal (*n* = 21)	NSIP (*n* = 11)	UIP (*n* = 31)	NSIP + UIP vs. Normal^a^	NSIP vs. Normal^b^	UIP vs. Normal^b^	UIP vs. NSIP^b^
*s* ^*^	0.21 ± 0.06	0.22 ± 0.07	0.17 ± 0.08	0.048	0.920	0.030	0.040
*s* ^*^, upper	0.10 ± 0.03	0.15 ± 0.05	0.10 ± 0.06	0.770	0.140	0.830	0.037
*s* ^*^, lower	0.29 ± 0.09	0.32 ± 0.11	0.23 ± 0.11	0.015	1.000	0.009	0.038
*s* ^*^_yz_	0.20 ± 0.06	0.21 ± 0.07	0.15 ± 0.07	0.031	0.950	0.018	0.032
*s* ^*^_yz_, upper	0.10 ± 0.03	0.14 ± 0.05	0.09 ± 0.06	0.950	0.140	0.690	0.024
*s* ^*^_yz_, lower	0.27 ± 0.09	0.29 ± 0.11	0.20 ± 0.10	0.012	1.000	0.007	0.036
*θ*	37.6 ± 13.5	33.7 ± 14.8	23.7 ± 18.9	0.008	NA	0.012	NA
*θ*, upper	17.6 ± 20.5	22.0 ± 17.1	12.4 ± 20.8	0.630	NA	NA	NA
*θ*, lower	52.9 ± 10.3	47.4 ± 16.9	32.4 ± 21.9	< 0.001	0.680	< 0.001	NA

a *p* –values of Student’s t-test.

b *p* –values of ANOVA post hoc test (NA denotes p > 0.05 by ANOVA).

Values in columns 2–4 are means ± SD. Values in columns 5–8 are the *p* values.

*s* ^*^: 3D displacement magnitude calculated from the x, y, and z components.

*s* ^*^_yz_: DB displacement magnitude from the y and z components indicatinghe tdorsal and basal lung motion.

upper: upper and middle lobes; lower: lower lobes

### 3.4 Tissue fractions

**FIGURE 4:**
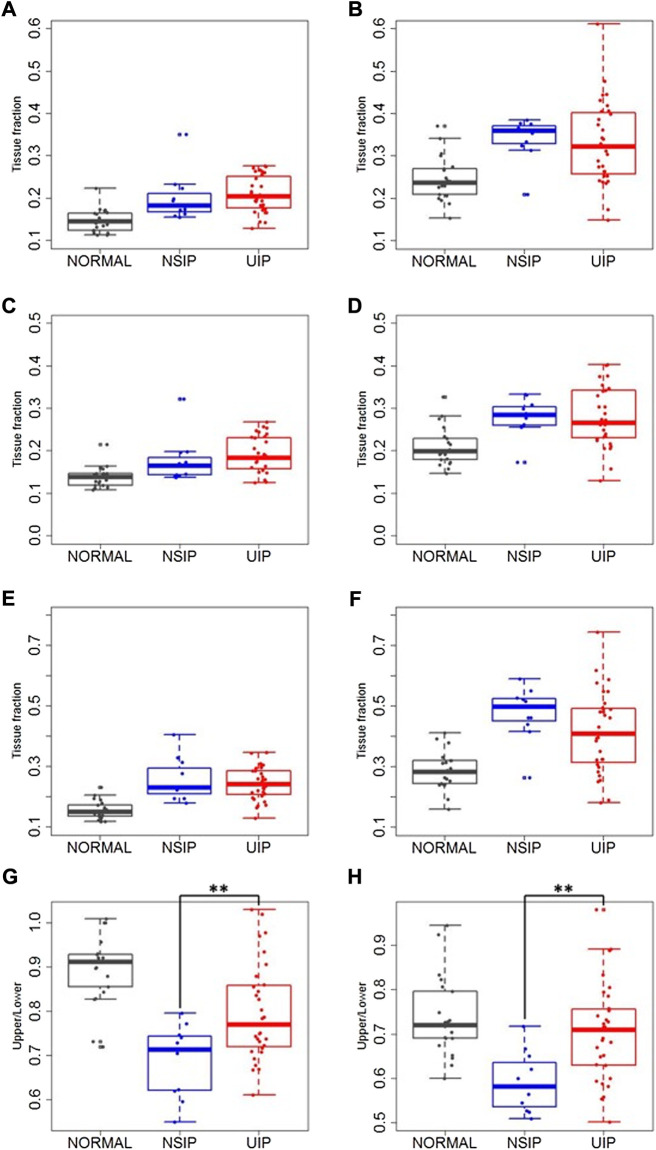
Local TFs on **(A,C,E,G)** inspiration and on **(B,D,F,H)** expiration in the whole lung **(A,B)**, upper and middle lobes **(C,D)**, lower lobes **(E,F)**, and upper–lower ratio **(G,H)** in normal, NSIP, and UIP subjects. ** denotes *p* < 0.05.

Mean local TFs were also compared (Table 3 and Figure 4). TFs increased in ILD (both NSIP and UIP) patients compared to those in normal subjects in the upper and the lower lobes and consequently in the whole lung on both inspiration and expiration (*p* < 0.001 for all). Between NSIP and UIP, TFs do not significantly differ in all individual lung regions. However, the upper lobe to lower lobe ratio is significantly smaller in NSIP than in UIP and also than normal subjects both on inspiration (*p* = 0.002) and on expiration (*p* < 0.001). The upper–lower ratio is significantly smaller in IPF than normal subjects on inspiration (*p* < 0.001) but not on expiration (*p* = 0.240). In NSIP, the upper–lower ratio of local TF is significantly smaller than normal subjects both on inspiration (*p* < 0.001) and expiration (*p* < 0.001).

**TABLE 3:** Comparison of TFs between normal, NSIP, and UIP patients.

	Normal (*n* = 21)	NSIP (*n* = 11)	UIP (*n* = 31)	NSIP + UIP vs. Normal^b^	NSIP vs. Normal^b^	UIP vs. Normal^b^	UIP vs. NSIP^b^
IN, all lobes (%)	14.6 ± 2.7	20.0 ± 5.6	20.9 ± 4.3	< 0.001	0.011	< 0.001	0.660
IN, upper lobes (%)	13.8 ± 2.5	17.5 ± 5.3	19.1 ± 4.1	< 0.001	0.050	< 0.001	0.380
IN, lower lobes (%)	15.6 ± 3.1	25.5 ± 6.9	24.3 ± 5.4	< 0.001	< 0.001	< 0.001	0.590
IN, upper/lower lobes	0.896 ± 0.076	0.690 ± 0.080	0.797 ± 0.109	< 0.001	< 0.001	< 0.001	0.002
EX, all lobes (%)	24.6 ± 5.2	34.1 ± 4.9	33.1 ± 9.8	< 0.001	< 0.001	< 0.001	0.640
EX, upper lobes (%)	20.9 ± 4.5	27.9 ± 4.5	28.5 ± 8.3	< 0.001	< 0.001	< 0.001	0.770
EX, lower lobes (%)	28.6 ± 6.2	47.7 ± 8.7	41.5 ± 13.4	< 0.001	< 0.001	< 0.001	0.094
EX, upper/lower lobes	0.737 ± 0.090	0.591 ± 0.067	0.704 ± 0.108	0.020	< 0.001	0.240	< 0.001

a *p* –values of Student’s t-test.

b *p* –values of ANOVA post hoc test.

Values in columns 2–4 are means ± SD. Values in columns 5–8 are the *p* values.

### 3.5 Correlation between displacement and tissue fraction

Correlations of the TFs with displacements (3D and DB) were analyzed in normal subjects, NSIP, UIP, ILD (UIP + NSIP), and the entire subjects. In normal subjects, TFs on expiration showed high positive correlations with both 3D and DB displacements in all regions (Table 4). The correlations were slightly higher with 3D displacement (*r* = 0.72 for the whole lung) than with DB displacement (*r* = 0.71 for the whole lung). In four lobes, the left upper lobe (LUL), the right upper lobe (RUL), the right middle lobe (RML), and the right lower lobe (RLL), the correlations range between 0.68 and 0.71, while it is relatively lower in the left lower lobe (LLL) (*r* = 0.51) than the other lobes. Correlations between upper–lower ratios of TF and displacement (3D and DB) also show positive correlation (*r* = 0.59 and *r* = 0.58, respectively). On inspiration, no significant correlation between TFs and displacements was found in any region. Compared to the normal subjects, the correlations decreased in UIP lungs, particurly in the RML (*r* = 0.71 and *r* = 0.42 in normal and UIP, respectively, with 3D displacement). In contrast to normal subjects, the correlation with DB displacement became greater than with 3D displacement in all five lobes of UIP patients. Upper–lower ratios were not linearly correlated with displacements in either UIP or NSIP.

**TABLE 4:** Regional correlation of TF with 3D and dorso-basal displacements.

	Normal, 3D	Normal, DB	UIP, 3D	UIP, DB
LUL	0.71	0.69	0.62	0.66
LLL	0.51	0.5	0.46	0.47
RUL	0.68	0.64	0.54	0.62
RML	0.71	0.7	0.42	0.47
RLL	0.7	0.69	0.6	0.62
Upper lobes	0.75	0.73	0.58	0.64
Lower lobes	0.66	0.65	0.56	0.57
All lobes	0.72	0.71	0.59	0.62
upper/lower lobes	0.59	0.58	0.06	−0.05

Values are Pearson’s correlation coefficients.

## 4 Discussion

This study shows that imaging-based regional lung DB displacement can distinguish mechanical behaviors between UIP and NSIP. The gross measurement of the degree or extent of fibrosis on the CT scan could be an important biomarker for the assessment of idiopathic interstitial pneumonia. Although honeycombing on CT is one of the prognostic factors in IPF, the concordance rate between radiologists is not high (Watadani et al., 2013). Additionally, the high-resolution CT (HRCT) scan alone does not accurately reveal micro-honeycombing that could be one of the findings to make a diagnosis as UIP classified as a possible UIP on the CT scan (Travis et al., 2013). The lung motionography using the cross-volume imaging registration technique used in the present study may be one of the unique methods to reflect the degree and extent of lung motion limitations, which may enable the detection of the early stage of fibrosis and reflect the whole lung fibrosis or restricted lung function for the assessment of interstitial fibrotic lung disease.

### 4.1 Normalized displacement

CT findings of ILD could be affected by the lung volumes at the level of inspiration and expiration. In a clinical environment, inflation levels at which inspiration and expiration CT images are acquired may vary for many reasons, which could add uncertainty to the CT reading of individual images or quantitative analysis of changes between the two images. We propose a normalization of displacement by a cubic root of the lung volume change between two images, to minimize the effect of uncertainty due to lung inflation level, and to conduct standardized relative regional characteristics of local lung motion. We used the length scale for normalization to nondimensionalize the measure. The parenchymal unit volume to measure the local lung motion is at an acinar scale, which may reflect characteristics at the scale of the functional unit of the lung in addition to the smooth possible noise of information from smaller voxels. It has been shown that the normalization of image-registration-based regional lung functions using the global lung volumes has shown successful inter-subject quantitative CT analysis (Chae et al., 2020; Choi S et al., 2017; Shin et al., 2020; Kang et al., 2021).

### 4.2 Lower lobe functional limitation

As expected, the limitation of motion in the lower lobes corresponded to the distribution of honeycombing on CT scan. Dorsal-basal movement of the lung in the supine position for the CT scan is highest in the normal subjects and accordingly the dorsal-basal movement of the lung was mostly restricted in the patients with ILD. In addition to the motion limitation, the air volume change also decreased in the lower lobes in patients with ILD. All the patients were scanned in the supine position in this study. However, the lung motion and air volume change between inspiration and expiration in prone position could be interesting because the prone position improves arterial oxygenation, reduces shunts, and can affect diaphragmatic motions (Albert et al., 1987; Tomita et al., 2004). It is speculated that the increased stiffness due to excessive fibrosis with honeycombing limited regional lung motions in the lower lobes of UIP patients compared to normal and even to NSIP, as demonstrated in Figure 2 **.**

### 4.3 Direction of lung movement

The motivation of using 3D and 2D displacements originated from the idea that the degree of fibrosis in the lung may be associated with the reduced capability of lung motion, because regional lung motion is based upon the cumulative deformation of local lung regions, which are limited in the restrictive changes in UIP and NSIP. Since the 3D volumetric analysis is available and the nature of lung deformation is three-dimensional, we used 3D displacement. Considering that the regional lung deformation primarily varies through dorsoventral and apicobasal axes, but not necessarily through the lateromedial axis, with gravitational dependency, we used 2D formulation as well.

From normal to NSIP and UIP, the direction of the lung movement also changes from the posterior costophrenic angle direction to the anterior costophrenic angle direction with the deformation of the lung morphology. This may mainly be attributable to the distribution of honeycombing or lung fibrosis in the dorsal and basal regions of the lung. As demonstrated in Figure 2, the proposed method can illustrate the morphologic deformation of the whole lung in patients with restricted lung function compared to the normal subjects.

### 4.4 Association with pulmonary function test analysis

Among the PFT results presented in Table 1, only FVC (%pred) differentiated UIP against NSIP. However, other measures including DL_CO_ were not significantly different between UIP and NSIP. This may imply that the earlier stage of fibrosis, septal thickening, or inflammatory process may all contribute to a decrease in DL_CO_, and the progression of fibrosis decreases the lung motion, in association with the decline of the full ventilation capacity (decreased FVC). A lower lobe predominance of air volume change between inspiration and expiration also supports this argument. With this interpretation, the proposed CT-based lung motionography is considered useful for capturing the progression of the fibrotic disease, since the contribution of CT imaging in the characterization of IPF is gaining more attention and acceptance in clinical use (Raghu et al., 2018, 2022) and the proposed method provides regional alteration of lung motion at such small scales as acini or voxels.

### 4.5 Tissue fraction

Regional TF analysis results support the above discussion. Regional characteristics of local TFs on inspiration and on expiration can be interpreted as indicators of small-scale structural alteration, and regional characteristics of local displacements serve as indicators of small-scale functional alteration. We speculated that the increase of TF seen in NSIP might be explained by the structural alteration with the early stage of fibrosis and relatively lower functional decrease compensated by regional hyperinflations in contrast to UIP with advanced fibrosis and restricted lung function. In this study, the result of increased expiratory TF in UIP is also in agreement with the report of a previous study (Petroulia et al., 2018). The correlation of the results between displacements and TFs suggests the following interpretation. In normal subjects, the positive correlation of displacements only with expiratory TF and not with inspiratory TF reflects deformation characteristics of healthy lungs. On inspiration near the total lung capacity (TLC), TFs are relatively uniform due to the full recruitment of alveoli; the deflation from TLC is greater in more dorsal and basal regions along with more displacement and more expiratory TFs (less air fractions) in these regions. This may imply that the mechanical property of local lung structure is presumably homogeneous in normal subjects. TF increase in NSIP and UIP patients may indicate the presence of fibrosis or thickening of the interlobular septum and/or intralobular septum in the lung parenchyma. TF was better correlated with DB displacement than 3D displacement in all five lobes of UIP patients. This supports that DB displacement may better characterize the UIP-associated structural alteration such as fibrosis or intra- and interlobular septal thickening than 3D displacement.

### 4.6 Study limitations

One of the limitations of the current study is that the inspiration to expiration volume change could be affected by the level of respiration of the patients. This may limit the accurate assessment of the ILD. However, the disease itself may manifest a decreased volume change between inspiration and expiration and also due to the decreased lung volume with fibrosis. We tried to minimize the effect of variability in lung volumes between inspiration and expiration. Further studies may be needed to better understand the relationship of the degree of fibrosis and lung volumes and to compare normalization based on other quantities, which requires more subjects. The clinically diagnosed NSIP group without CT honeycombing may have pathological UIP with micro-honeycombing or atypical UIP. But the purpose of this study was to assess the lung motion related to the degree of fibrosis presented as honeycombing on CT scan, which represents a more advanced stage of interstitial fibrosis compared to reticular opacity or ground-glass opacity on CT scan.

The focus of the current study is limited to the discriminative characterization of lung motionography between UIP and NSIP. Characterization of the associated nature between impaired lung motionography with common clinical measures including PFTs, such as linear or nonlinear relationship, is not fully understood and remains for future study with a greater sample size. The scope of the current work does not include the connected tissue disease (CTD)-associated ILD. It may be worth applying the regional lung motionography analysis for RA-ILD, SSc-ILD, and Sjogren disease, which remains for future studies. Also, the methods for the analysis of lung motion in the current study is limited to CT-based approaches. However, further investigation of lung motion in ILD associating with other radiological methods such as ultrasound B-lines (Manolescu et al., 2020, Tardella et al., 2018) may provide additional understanding of impaired lung motionography that can expand the lung motion assessment for more clinical applications.

## 4.7 Concluding remarks

In conclusion, CT-based regional lung motionography may be used to illustrate the restricted lung motion or indirectly reflect the degree or extent of lung fibrosis. Lower lobe lung motions using image registration technique were significantly different between normal, NSIP, and UIP patterns on CT scans.

## Data Availability

The raw data supporting the conclusions of this article will be made available by the authors without undue reservation, under consideration of the terms that participants were not asked for consent to make individual data available. Requests to access the datasets should be directed to changhyun.lee@snu.ac.kr.

## Abbreviations

IPF, idiopathic pulmonary fibrosis; CT, computed tomography; FVC, forced vital capacity; DL_CO_, diffusion capacity of carbon monoxide; MRI, magnetic resonance imaging; COPD, chronic obstructive pulmonary disease; ILD, interstitial lung disease; UIP, usual interstitial pneumonia; NSIP, nonspecific interstitial pneumonia; ATS, American Thoracic Society; ERS, European Respiratory Society; GGO, ground glass opacity; 3D, three-dimensional; PFT, pulmonary function test; FEV_1_, forced expiratory volume in 1 s; SSTVD, sum of squared tissue volume difference; 2D, two-dimensional; DB, dorso-basal; *s**, normalized 3D local displacement magnitude; *s*, 3D local displacement magnitude; V^*IN*^, inspiratory lung volume; V*^EX^*, expiratory lung volume; *x^IN^*, inspiratory position of the local lung region; *x^EX^*, expiratory position of the local lung region; θ, displacement angle; LAA, low attenuation area; HU, Hounsfield unit; HRCT, high-resolution CT.
