# Supplementary figures and images for: CT-based lung motion differences in patients with usual interstitial pneumonia and nonspecific interstitial pneumonia
Source: Front Physiol. 2022 Oct 4;13:867473. doi: 10.3389/fphys.2022.867473 (PMC9577177; doi:10.3389/fphys.2022.867473)

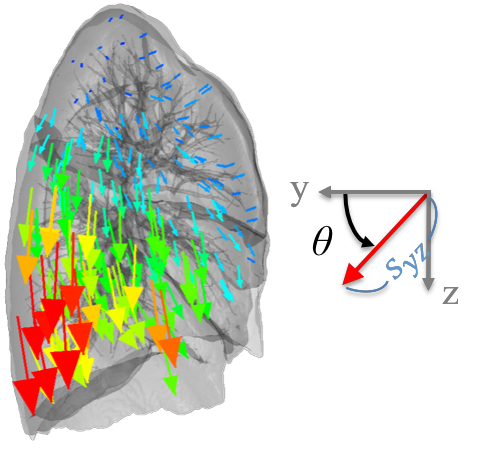

Supplement: Supplementary file 1 [file Image3.TIFF]

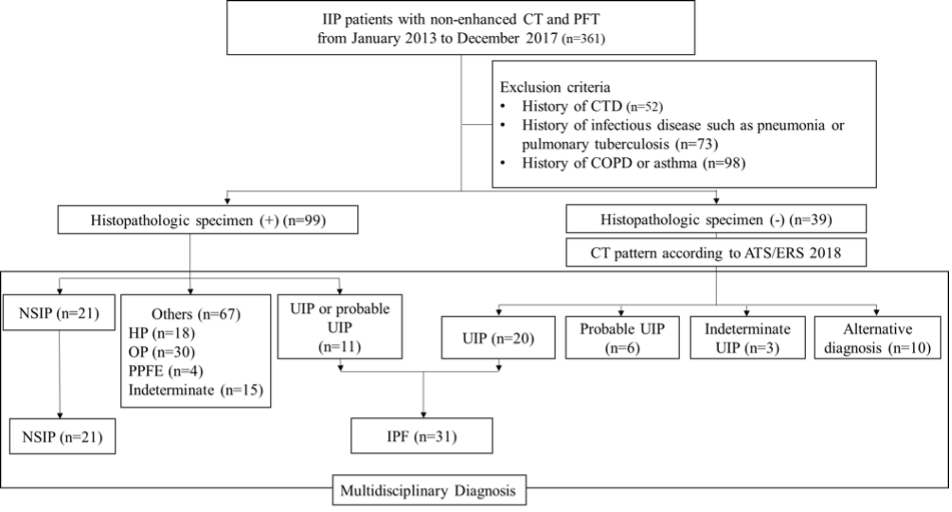

Supplement: Supplementary file 2 [file Image1.TIFF]

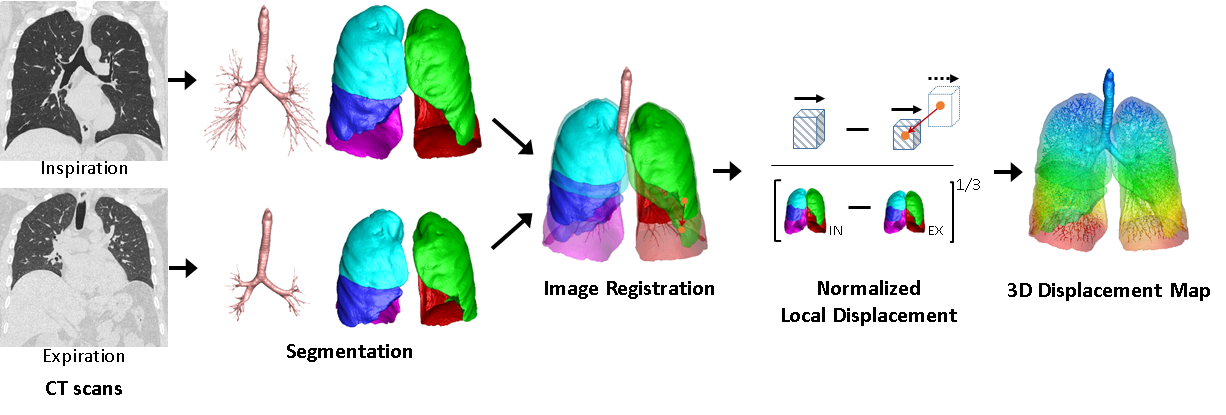

Supplement: Supplementary file 4 [file Image2.TIFF]
