# Supplementary material for: CT-based lung motion differences in patients with usual interstitial pneumonia and nonspecific interstitial pneumonia
Source: Front Physiol. 2022 Oct 4;13:867473. doi: 10.3389/fphys.2022.867473 (PMC9577177; doi:10.3389/fphys.2022.867473)
Supplement: Supplementary file 3 [file Table1.docx]

Supplementary Material

# Supplementary Figures


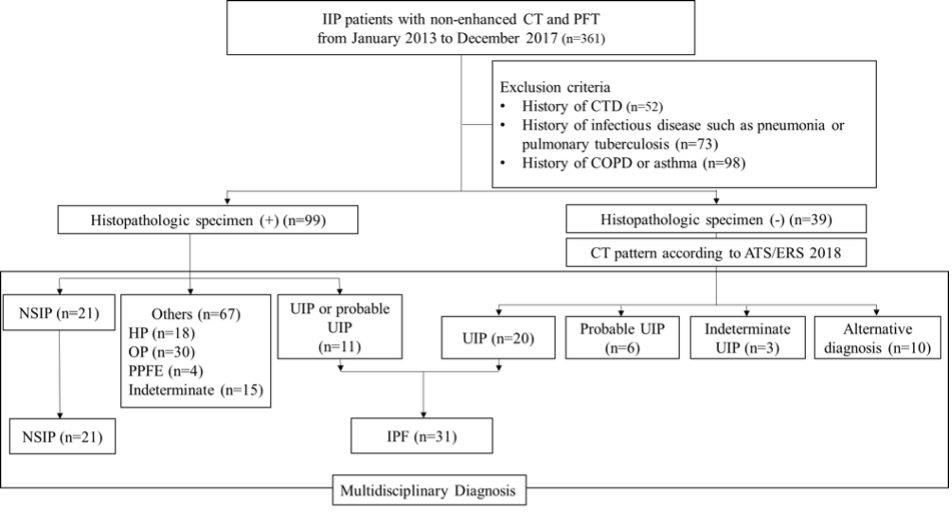


**Supplementary Figure 1.** Flow diagram of included patients.

**
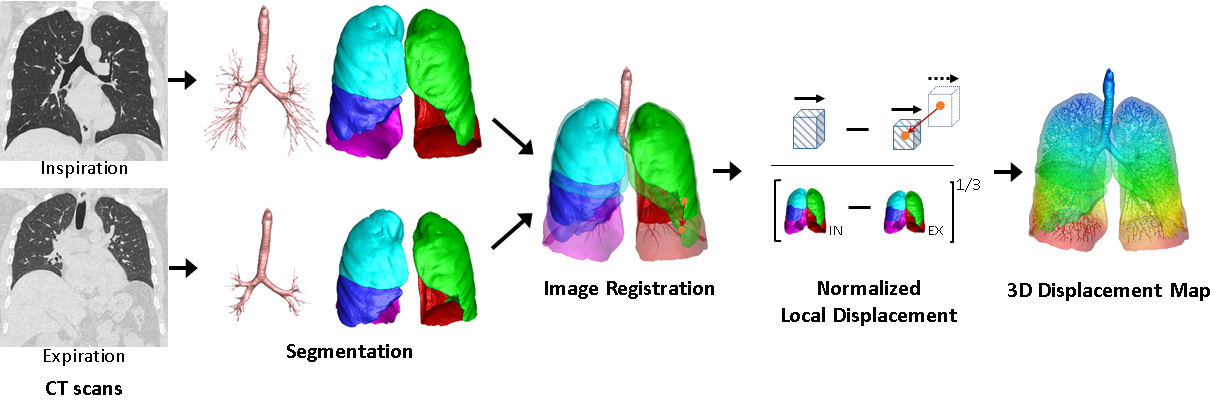
**

**Supplementary Figure 2.** Schematic diagram of workflow from inspiration and expiration CT scans to the local lung displacement map.

**
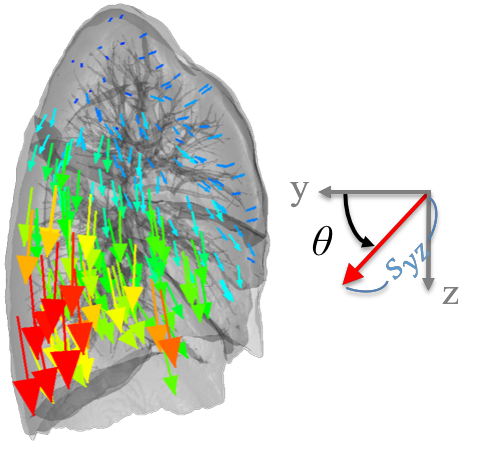
**

**Supplementary Figure 3.** Lateral view of 3D displacement vectors and a schematic of θ and 𝑠_𝑦𝑧_.
